# Draft Genome Sequence of *Bacillus vietnamensis* Strain UCD-SED5 (Phylum *Firmicutes*)

**DOI:** 10.1128/genomeA.01376-15

**Published:** 2015-11-19

**Authors:** Ruth D. Lee, Guillaume Jospin, Jenna M. Lang, Jonathan A. Eisen, David A. Coil

**Affiliations:** a University of California Davis Genome Center, Davis, California, USA; b University of California Davis, Department of Evolution and Ecology, Department of Medical Microbiology and Immunology, Davis, California, USA

## Abstract

Here, we present the draft genome sequence of *Bacillus vietnamensis* UCD-SED5 (phylum *Firmicutes*). This strain was isolated from sediment surrounding *Zostera marina* roots near the UC Davis Bodega Marine Laboratory (Bodega, Bay, California) and represents the second genome of this species. The assembly consists of 4,325,707 bp, in 108 contigs.

## GENOME ANNOUNCEMENT

*Bacillus vietnamensis* UCD-SED5 was isolated from sediment surrounding common eelgrass (*Zostera marina*) roots near the UC, Davis Bodega Marine Laboratory (Bodega Bay, California, USA). The sampling site was located north of Westshore Park, California (38°19'10.0"N, 123°03'13.8"W).

*B. vietnamensis* was originally isolated from fermented fish consumables, a common food used in Asian cuisine, and was shown to be halotolerant (1). Previous studies have also claimed that the genus *Bacillus* plays a large and necessary role in the fermentation of fish products (1).

Dilutions of sediment in lysogeny broth (LB) of 1:100 and 1:1000 were made and spread on LB plates, grown at room temperature for 24 h, and individual colonies were double dilution struck. A Wizard Genomic DNA purification kit (Promega) was used to extract DNA from a fresh 5-mL overnight culture. Sanger sequencing was performed on the amplified 16S rRNA PCR products (27F: AGAGTTTGATCMTGGCTCAG and 1391R: GACGGGCGGTGTGTRCA). Examination of BLAST search results and phylogenetic analyses (2) suggested identity to either *Bacillus vietnamensis* or *Bacillus aquimaris*. BLAST results were both >99% identical and the 16S phylogeny was ambiguous. Because *B. vietnamensis* can grow well outside the pH range of *B. aquimaris* (1, 3), we tested media at various pH, and this suggested the identification as *B. vietnamensis*. Hopefully, the addition of more genome sequences from both species will allow better phylogenetic resolution between the two groups, if such a division even exists.

A Nextera DNA sample prep kit (Illumina) was used to make a paired-end library (Illumina). Libraries were sequenced on an Illumina MiSeq, at a read length of 300 bp. A total of 883,585 high-quality paired-end reads was processed by the A5 assembly pipeline (4). This pipeline automates data cleaning, error correction, contig assembly, quality control, and scaffolding. The resulting assembly consisted of 108 contigs (longest: 618,024 bp; *N*_50_: 287,494) that were submitted to GenBank. This final assembly had 4,325,707 bp with a G+C content of 43.5% and an overall coverage estimate of ~102×. Genome completeness was assessed using the PhyloSift software (5), which searches for a list of 37 highly conserved, single-copy marker genes (6), of which all 37 were found in this assembly.

The RAST server was used to perform an automated annotation (7–9). *B. vietnamensis* UCD-SED5 contains 4,470 predicted protein-coding sequences and 145 predicted noncoding RNAs.

### Nucleotide sequence accession numbers.

This whole-genome shotgun project has been deposited at DDBJ/EMBL/GenBank under the accession no. LIXZ00000000. The version described in this paper is version LIXZ01000000.
